# Metformin and cancer hallmarks: shedding new lights on therapeutic repurposing

**DOI:** 10.1186/s12967-023-04263-8

**Published:** 2023-06-21

**Authors:** Yu Hua, Yue Zheng, Yiran Yao, Renbing Jia, Shengfang Ge, Ai Zhuang

**Affiliations:** 1 Department of Ophthalmology, Ninth People’s Hospital, Shanghai Jiao Tong University School of Medicine, No. 639 Zhizaoju Road, Shanghai, 200011 China; 2 Shanghai Key Laboratory of Orbital Diseases and Ocular Oncology, No. 639 Zhizaoju Road, Shanghai, 200011 China

**Keywords:** Metformin, Cancer, Hallmarks, Mechanisms

## Abstract

Metformin is a well-known anti-diabetic drug that has been repurposed for several emerging applications, including as an anti-cancer agent. It boasts the distinct advantages of an excellent safety and tolerability profile and high cost-effectiveness at less than one US dollar per daily dose. Epidemiological evidence reveals that metformin reduces the risk of cancer and decreases cancer-related mortality in patients with diabetes; however, the exact mechanisms are not well understood. Energy metabolism may be central to the mechanism of action. Based on altering whole-body energy metabolism or cellular state, metformin’s modes of action can be divided into two broad, non-mutually exclusive categories: “direct effects”, which induce a direct effect on cancer cells, independent of blood glucose and insulin levels, and “indirect effects” that arise from systemic metabolic changes depending on blood glucose and insulin levels. In this review, we summarize an updated account of the current knowledge on metformin antitumor action, elaborate on the underlying mechanisms in terms of the hallmarks of cancer, and propose potential applications for repurposing metformin for cancer therapeutics.

## Introduction

Metformin is one of the most commonly prescribed anti-diabetic drugs worldwide. Its history can be traced back to 1918 when guanidine, found in traditional herbal medicine in Europe known as Galega officinalis, was shown to lower glycemia [1]. A series of guanidine derivatives, including metformin, was subsequently synthesized [2]. Over time, the benefits associated with repurposing metformin for several challenging diseases, including obesity [3], cardiovascular diseases [4, 5], liver diseases [6], renal diseases [7], aging-related diseases [8], and cancers [9] have been shown. Epidemiological studies have revealed that metformin exerts protective effects on people with diabetes suffering from cancer [10–12]. Intriguingly, several clinical studies have also reported encouraging outcomes in non-diabetic cancer patients [13–15]. Given that metformin is safe, well-tolerated, and cost-effective, it is extremely appealing as a focus of antitumor research. Subsequent evidence has shown that metformin inhibits tumor growth, invasion, and metastasis both in vitro and in mouse tumor models for hepatocellular carcinoma [16], ocular melanoma [17], head and neck squamous cell carcinoma [18], and breast cancer [19], among others. Moreover, metformin has been used as a synergistic therapy for cancer, as it enhances sensitivity to radiotherapy [15, 20], chemotherapy [14, 21], and immunotherapy [22] and decreases side effects at lower therapeutic dosages of anticancer treatments.

Great interest has been attached to the basic and clinical study of metformin in cancer. The central mechanism by which metformin attenuates tumorigenesis and progression is through the regulation of energy metabolism. The master pathway of metformin anticancer activity is the activation of the adenosine monophosphate-activated protein kinase (AMPK)/mammalian target of rapamycin (mTOR) pathway triggered by inhibition of complex I in the mitochondrial respiratory chain [23–25]. However, the vague performance in a clinical study was in contrast with the excellent performance in a preclinical study. Metformin did not show any benefit in cancer treatment in some clinical trials. Therefore, there are great challenges in the clinical translation of metformin.

**Fig. 1:**
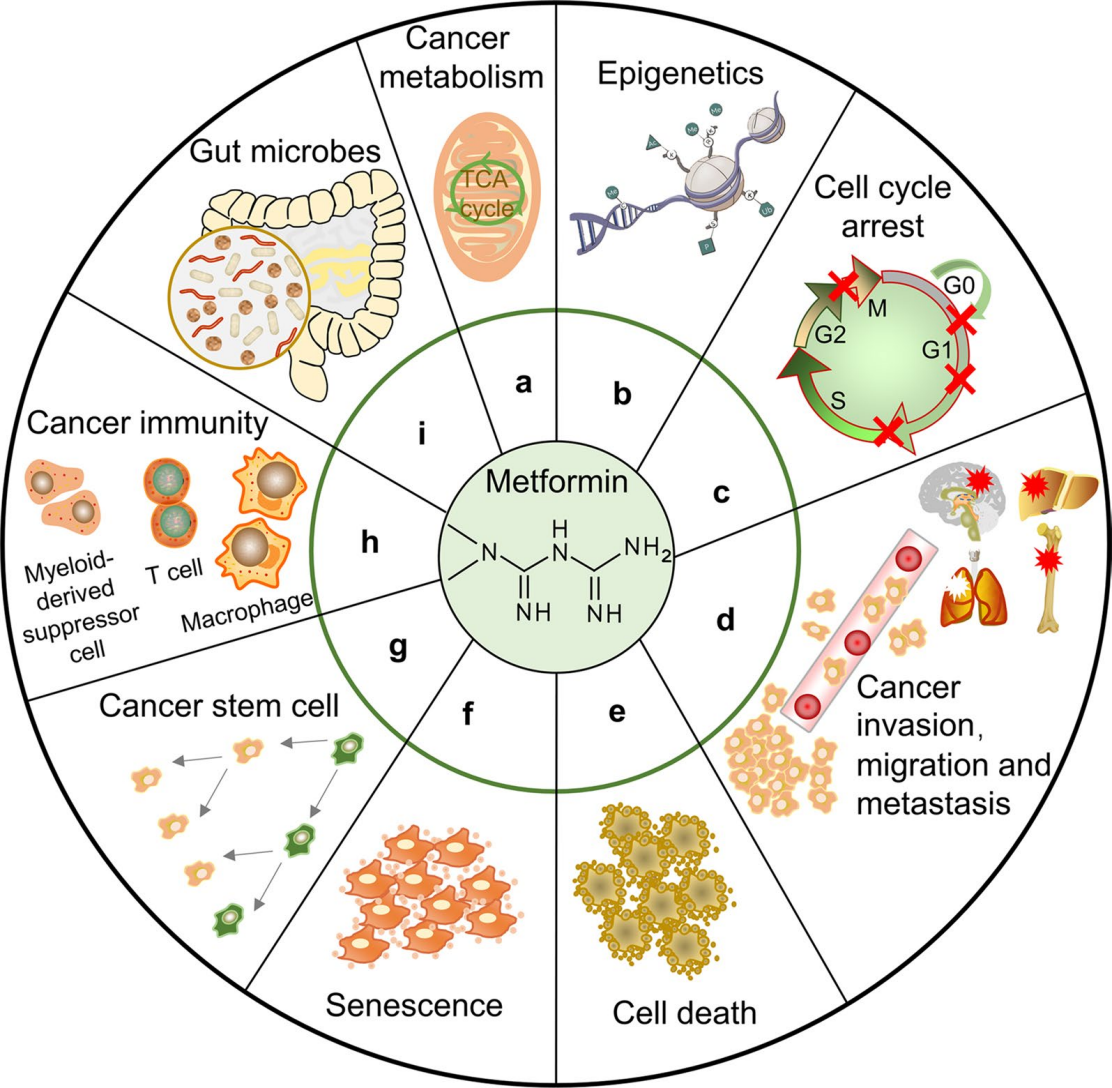
Main anticancer mechanisms of action of metformin based on hallmarks of cancer. Metformin can combat cancer by affecting metabolism, epigenetics, cell cycle, migration, metastasis, cell death, cell senescence, cancer stem cells, immunity, and gut microbes

Abundant reviews have elaborated on the topic of metformin and cancer from different perspectives, such as specific cancer types [26, 27], diabetes [28, 29], pharmacology [30, 31], and molecular mechanisms [32, 33]. However, insight into the therapeutic repurposing of metformin is still insufficient [34]. Based on the literature review, we recognize that metformin exerts protective effects against multiple tumor types and an increasing number of subtypes [35, 36]. Hence, the mechanisms of action of metformin must be closely related to the hallmarks of cancer [37], which have been proposed as a common set of functional capabilities crucial to the transformation from normalcy to malignancy. This review focused on the effects of metformin on cancer cells in terms of the hallmarks of cancer and updated the clinical translation of metformin in cancer treatment. In this review, we aim to (1) update the readers on the molecular mechanisms through which metformin exhibits antitumor activities, (2) map the effects of metformin on cancer cells in terms of the hallmarks of cancer (Fig. 1), and (3) summarize seminal clinical trials and therapeutic prospects of metformin for cancer treatment.

## Update on metformin’s molecular antitumor mechanisms of action

The classic modes of metformin’s antitumor effects are the inhibition of respiratory complex I in the mitochondria and the activation of AMPK in succession. Recently, metformin was defined to inhibit complex I by binding in the quinone channel and exert an independent localized chaotropic effect by combining cryo-electron microscopy and enzyme kinetics [38]. Although the direct interaction between metformin and complex I is essential, metformin-induced complex I inhibition is not a consequence of the direct interaction but instead occurs through an indirect mechanism [39]. Ma et al. [40] conducted another novel study that focused on direct molecular targets of metformin and identified PEN2, a subunit of γ-secretase, as a direct molecular target of metformin. PEN2 binds to ATP6AP1, inhibits the activity of v-ATPase without increasing AMP or ADP, and then activates the lysosomal AMP-independent AMPK pathway.

Epidemiologic studies indicate that metformin decreased the risk of cancer incidence compared to other anti-diabetic medications. Hence, the anticarcinogenic effects of metformin were traditionally divided into direct (blood glucose- and insulin-independent) and indirect (blood glucose- and insulin-dependent) effects, being mindful that none of the effects are mutually exclusive.

### Direct effects of metformin

Metformin can exert direct effects on cancer cells independent of blood glucose and insulin levels, partly through AMPK activation. It is generally acknowledged that metformin inhibits complex 1 (NADH-coenzyme Q oxidoreductase) of the mitochondrial respiratory chain, which leads to membrane depolarization, reactive oxygen species (ROS) release, and a decrease in the ATP/ADP ratio [41, 42]. Metformin requires a robust inner mitochondrial membrane potential to accumulate within the mitochondrial matrix and reversibly inhibits complex 1 [23]. This inhibition of complex I limits the electron flow to complex III, where ROS are generated. Mitochondrial complex III ROS are hypoxic activators of HIF-1 [43]. Therefore, metformin reduces the hypoxic stabilization of HIF-1α protein and HIF-dependent target genes. Additionally, metformin reduces DNA damage and the production of oxidative stress through mitochondrial respiratory chain inhibition [44]. Metformin depleted the tricarboxylic acid (TCA) cycle and blocked the production of biosynthetic precursors. Nearly all TCA cycle metabolites decrease considerably with metformin treatment [45]. Metformin can also inhibit cancer cell growth by decreasing the cellular energy status, and the effects can be reversed by the expression of the metformin-resistant yeast-derived complex I NADH dehydrogenase NDI1 [46].

**Fig. 2:**
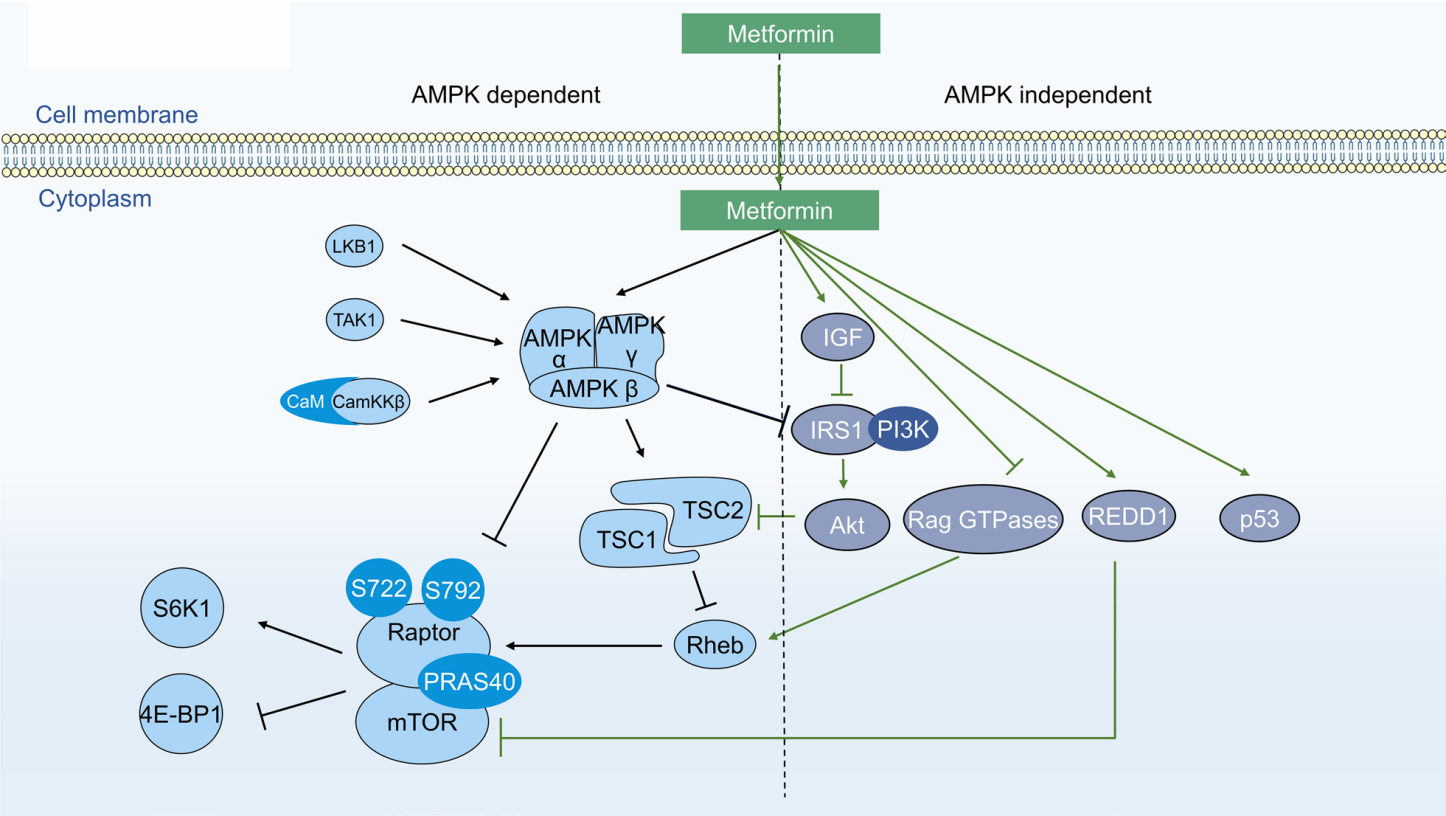
Main molecular anticancer mechanism of action of metformin. The pathways associated with anticancer action may be dependent on AMPK or independent of AMPK

A series of complicated signal pathways are activated by metformin (Fig. 2). First, metformin is a well-known AMPK activator and a key enzyme in glucose homeostasis, gluconeogenesis, and lipid metabolism. AMPK is directly activated by an increase in either the AMP/ATP or ADP/ATP ratio [47] and is indirectly activated by upstream kinases, including LKB1 [48], Ca(2+)/calmodulin-dependent protein kinase kinase (CaMKK) beta [49] and TGFβ-activated kinase-1 (TAK1) [50]. Wu et al. [51] recently demonstrated that metformin protects AMPK-mediated phosphorylation of serine 99, thus increasing TET2 stability and 5-hydroxymethylcytosine (5hmC) levels. A pathway linking diabetes to cancer was revealed through the definition of a novel ‘phospho-switch’ that regulates TET2 stability and a regulatory pathway that links glucose and AMPK to TET2 and 5hmC.

AMPK-dependent mTOR complex 1 (mTORC1) inhibition occurs via multiple downstream effectors that switch on ATP-producing processes and switch off ATP-consuming pathways [52]. These effects can be mediated by the activation of TSC1/TSC2 tumor suppressor genes [53, 54]. TSC1/2 can inhibit mTORC1 and the phosphorylation of its downstream effectors 4EBP1 and S6K [55]. mTORC1 inhibition also occurs as a result of the direct phosphorylation of S722 and S792 on Raptor, a vital mTORC1-binding partner [56].

Metformin can inhibit mTOR through Rag GTPase inactivation or REDD1 activation independent of AMPK activation. Metformin can also inhibit mTORC1 signaling independent of AMPK or TSC1/2, although it is dependent on Rag GTPases. Metformin inhibits growth by inhibiting the mitochondrial respiratory capacity, which inhibits the transit of the RagA-RagC GTPase heterodimer through the nuclear pore complex (NPC). A key transcriptional target, acyl-CoA dehydrogenase family member-10 (ACAD10), is activated when metformin induces the nuclear exclusion of the GTPase RagC, thereby inhibiting mTORC1 [57]. REDD1 (REgulated in Development and DNA damage responses 1), also known as RTP801, Dig2, or DDIT4, has been deemed a hypoxia-inducible factor-1 (HIF-1) target gene and plays a significant role in inhibiting mTORC1 signaling during hypoxic stress. Several other pathways are involved in the anticancer action of metformin, including PI3K/AKT/mTOR [58–60], K-Ras [61], nemo-like kinase (NLK) [62], c-Jun-N-terminal kinase (JNK) [63], and Stat3-Bcl-2 [64].

Recently, some studies revealed prognostic and predictive biomarkers, as well as a promising therapeutic target of metformin. Xie et al. [65] reported a better response prediction to metformin therapy in patients with increased glycerol-3-phosphate dehydrogenase 1 (GPD1) expression in 15 cancer cell lines. Moreover, GPD1 can enhance the anticancer activity of metformin by synergistically increasing the total inhibition of cellular glycerol-3-phosphate and inhibiting mitochondrial function. Besides, chloride intracellular channel-1 (CLIC1) was reported to boost proliferation, and its functional expression is required for metformin antineoplastic effects in glioblastomas [66, 67] and gallbladder cancer cells [68].

### Indirect effects of metformin

Metformin may also exhibit anti-cancer activity by reducing circulating glucose and insulin levels. Metformin, an insulin sensitizer, decreases plasma insulin and insulin-binding proteins, which can reduce insulin growth factor-1 (IGF-1) levels. In addition, AMPK activation by metformin can reduce the phosphorylation of insulin receptor substrate-1 (IRS-1), leading to a reduction in growth-promoting pathways, including the PI3K/AKT/mTOR signaling network [69, 70]. Hyperinsulinemia induces other indirect effects, such as reducing hepatic synthesis of sex-hormone-binding globulin, resulting in elevation of sex steroid hormones, which is associated with an increased risk of cancer development [71, 72]. Besides, hyperinsulinemia may activate chronic inflammation, which may promote tumorigenesis [73].

## The effects of metformin on the hallmarks of cancer

### Deregulation of cancer metabolism (Fig. 3)

**Fig. 3:**
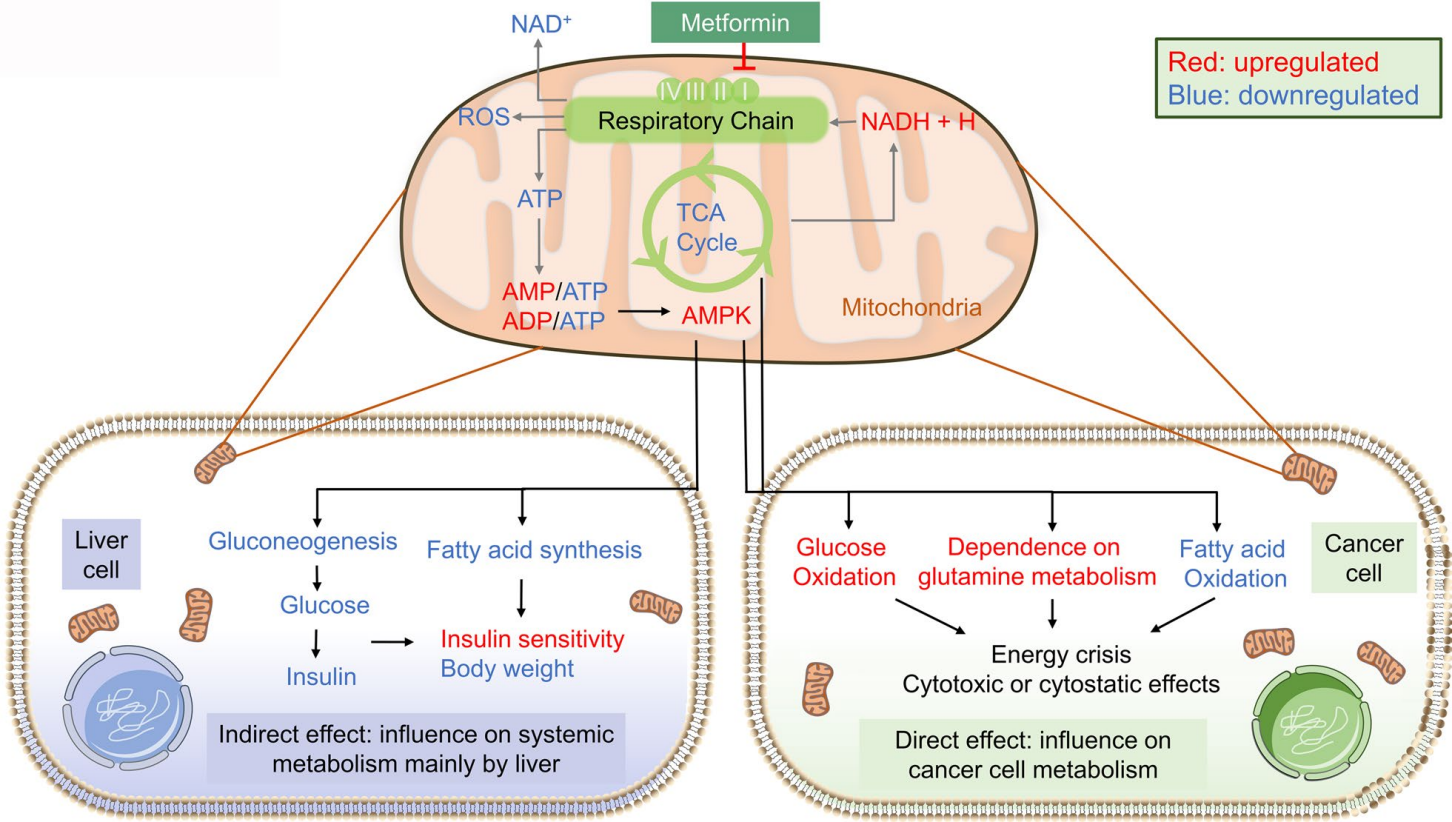
The primary site that at which metformin exerts its effects is the mitochondria. Metformin inhibits complex 1 in the respiratory chain and induces an elevation of AMP/ATP or ADP/ATP and subsequently activates AMPK. The indirect effects of metformin primarily rely on liver cells, where metformin decreases gluconeogenesis and fatty acid synthesis, thus influencing systemic metabolism. The direct effects of metabolism mainly involve the energy consumption of cancer cells

Metformin, originally discovered as an anti-diabetic agent, naturally plays a significant role in metabolism. The master pathways of metformin activity center on metabolism. Accordingly, the reprogramming of energy metabolism is an emerging hallmark of cancer. Cancer cells demand higher rates of catabolite uptake, transfer, and utilization than normal cells. Warburg [74] discovered that cancer cells alter metabolism with high rates of glucose uptake and increased lactate from glycolysis even under aerobic conditions, which is known as aerobic glycolysis. In this respect, metformin deserves further research as an anticancer drug because it mimics caloric restriction at both cellular and systemic levels.

Generally, metformin decreases glucose oxidation, increases dependency on reductive glutamine metabolism in cancer cells, and decreases fatty acid oxidation (FAO) as a result of TCA cycle inhibition [75, 76]. Lord et al. [77] identified two groups of breast cancer metabolic responses to metformin, an oxidative phosphorylation transcriptional response (OTR) group, in which there is an increase in oxidative phosphorylation (OXPHOS) gene transcription, and a fluorodeoxyglucose (FDG) response group with increased 18-FDG uptake. Besides, metformin can result in the concomitant acidification of the intra- and extracellular environment by modulating lactate metabolism [78]. Moreover, the acidification of the tumor microenvironment (TME) made tumors more susceptible to metformin due to the loss of NAD+ regeneration capacity [79]. Gui et al. [80] demonstrated that the inhibition of cancer cell proliferation by metformin is partly due to the loss of mitochondrial NAD+/NADH homeostasis and the inhibition of aspartate biosynthesis, which can be influenced by environmental factors. To target tumor metabolism, Elgendy et al. [81] proposed that the combination of intermittent fasting-induced hypoglycemia and metformin impairs tumor metabolic plasticity and growth via the PP2A-GSK3β-MCL-1 axis.

In addition to its effects on cancer cell metabolism, metformin also inhibits cancer through systemic metabolic changes. Vast evidence has revealed a relationship between cancer and metabolic disturbances, including diabetes mellitus [82–84]. Mechanistically, glucose, insulin, and insulin-like growth factors (IGFs) can promote cancer cell growth and progression, especially in insulin-sensitive cancers, which are where the indirect effects of metformin are based. Metformin decreases hepatic glucose output, leading to systemic glucose reduction and an improvement in secondary hyperinsulinemia, indirectly inhibiting cancer cell proliferation without accumulation in cancer cells.

The crucial role of energy metabolism implies that metformin may hold therapeutic value in cancer.

### Effects on epigenetics

Epigenetics is the study of heritable phenotypic changes that do not involve alterations in the DNA sequence and play an essential role in the differentiation of numerous types of cells, including cancer cells. Mounting evidence supports the high responsiveness of epigenetic regulatory machinery to metabolic cues [85, 86], and the antitumor mechanism of action of metformin involves the metabolic regulation of epigenetics.

Metformin regulates DNA methylation in cancer cells through mitochondrial one-carbon metabolism and histone acetylation. S-adenosylmethionine (SAM) is a universal methyl group donor for cellular methylation, and S-adenosylhomocysteine (SAH) is a feedback inhibitor of SAM-dependent DNA methyltransferases. Metformin was shown to induce global DNA methylation changes mediated by the H19/adenosylhomocysteine hydrolase (SAHH) axis [87]. Subsequently, Cuyàs et al. reported that metformin can increase the SAM:SAH ratio-related methylation capacity by targeting the coupling between serine mitochondrial one-carbon/CI flux [88]. In addition, the mutation or overexpression of EZH2 (H3K27-methyltransferase) has been linked to cancer [89] and several studies have demonstrated that metformin may affect EZH2 or H3K27-methylation [90–93]. As an AMPK activator, metformin may directly phosphorylate EZH2 at Thr311 to disrupt its interaction with SUZ12 and inhibit PRC2 methyltransferase activity and oncogenic function [90]. Metformin has been shown to combat EZH2-high prostate cancer by stimulating SETD2, which regulates EZH2-K735me1 to induce EZH2 destruction, thereby inhibiting prostate cancer metastasis [91]. In terms of acetylation, metformin inhibits SMAD3 phosphorylation and hinders the KAT5-SMAD3 interaction, which reduces KAT5-mediated K333 acetylation of SMAD3 to inhibit SMAD3 transcription and TRIB3 expression, thereby restoring autophagy and combating melanoma progression [94]. Metformin also inhibits the progression of ocular melanoma by inhibiting autophagy through histone deacetylation of optineurin (OPTN), a key candidate for autophagosome formation and maturation [17]. Additionally, metformin has been found to distinctly alter the expression of various microRNAs (miRNAs) in breast cancer [95], lung cancer [96], pancreatic cancer [97], renal cell carcinoma [98], and esophageal carcinoma [99]. Mechanistically, miRNAs are influenced by the expression of DICER, a key enzyme that processes miRNAs, thereby affecting gene expression patterns [100].

### Effects on cell proliferation, malignancy progression, and death

#### Suppression of cancer proliferation

The cell cycle can regulate cancer progression by sustaining proliferative signaling, a hallmark of cancer. Another anticancer role that metformin is considered to play is the induction of cell cycle arrest. Metformin induces cell cycle arrest at different stages, depending on the cancer type. For example, metformin induces cell cycle arrest at the G0/G1 stage in lung cancer cells [101], gastric cancer cells [102], hepatocellular carcinoma cells [103], osteosarcoma stem cells [104] and myeloma cells [60, 105]; G1 stage in breast cancer [106], thyroid cancer [107], bladder cancer [108] and castration-resistant prostate cancer (CRPC) cells [109]; G1/S stage in gastric cancer [110]; and G2/M stage in ovarian cancer [58], osteosarcoma [63], and melanoma cells [111].

Cell cycle arrest is mediated by various mechanisms, such as the activation of tyrosine phosphatases, interference with the MAP kinase ERK pathway, induction of cyclin-dependent kinase inhibitors, and accumulation of hypophosphorylated Rb protein. In colorectal cancer cells, metformin has been reported to induce cell cycle arrest in the G0/G1 phase and reduce the expression of CycD1 and c-Myc and the phosphorylation of Rb (a CycD1 downstream target) [112]. Kato et al. [102] showed that metformin induces cell cycle arrest in the G1/S phase, increases the expression of GADD45 (a stress sensor and a cell cycle regulator), increases the expression of P21 (a cyclin-dependent kinase inhibitor), decreases the expression of E2F1 (a pro-proliferating transcription factor), and decreases proliferating cell nuclear antigen (PCNA) in gastric cancer cells. Zimmermann et al. [106] discovered a synergistic effect between metformin and fulvestrant, an estrogen receptor (ER) antagonist, on cell cycle arrest in ER-positive breast cancer cells. Metformin enhances CycG2 expression and potentiates the CycG2 expression and cell cycle arrest induced by fulvestrant. Elevation of CycG2 is required to induce G1-phase cell cycle arrest triggered by the blockade of E2/ER signaling. In bladder cancer cells, metformin activates AMPKα, which promotes the degradation of Yes-associated protein 1 (Yap1), a key molecule of the Hippo pathway. The formation of the Yap1-TEADS4 complex positively regulates CCNE1 and CCNE2 expression. This regulation of the AMPKα/Yap1/TEAD4/CCNE1/2 axis makes blockading the cell cycle a potential anticancer mechanism of action for metformin [108]. Varghese et al. [113] demonstrated an association between glucose concentration and metformin efficacy in triple-negative breast cancer cells (TNBCs). Cell cycle arrest occurs at the G0/G1 phase under higher glucose conditions and the G2/M phase only under lower glucose conditions. Therefore, a combination of agents that inhibit the glycolytic pathway may be more beneficial for TNBC treatment. Additionally, the anti-cancer effect of metformin depends on the cell type and concentration of metformin.

Blockade of the cell cycle is an essential way to produce anticancer effects. Metformin has been shown to induce cell cycle arrest at different phases in various types of cancer, although it is most effective with the appropriate drug concentration, glucose concentration, and adjuvant therapy.

#### Abrogation of invasion and metastasis

Invasion and metastasis are hallmarks of cancer and key features leading to the high mortality associated with cancer. Metastasis is a multistep process that involves the migration and invasion of tumor cells into the stroma and blood or lymphatic vessels. Metalloproteinases (MMPs) play a critical role in cancer migration, invasion, and metastasis by degrading extracellular matrix proteins. In their study of HCC cell lines, Sun et al. [59] reported that metformin induced a reduction in MMP-9 expression and an inhibition of cancer cell invasion that was enhanced when combined with aloin. Ferretti et al. [114] demonstrated that metformin could suppress cell migration and invasion, although this was dependent on an intact AMPK-p53 axis and could be potentiated by glucose restriction. In addition, metformin increased E-cadherin, decreased vimentin, and inhibited epithelial-mesenchymal transition (EMT) in HCC cells. Likewise, metformin was found to inhibit EMT in chemoresistant rectal cancer cells by blocking transforming growth factor (TGF)-β receptor type 2 (TGFBR2)-mediated Snail and Twist expression [115]. Wang et al. [116] demonstrated that metformin inhibits lung metastasis through its vascular effects on metastatic breast cancer. Metformin remodels abnormal vessels (also known as “vessel normalization”), which reduces microvessel density, leakage, and hypoxia and increases vascular mural cell coverage and perfusion via downregulation of platelet-derived growth factor B (PDGF-B).

#### Promotion of cell death

##### Apoptosis

Apoptosis is vital to the normal activity of an organism, but inappropriate apoptosis is a factor in many diseases, including many types of cancer. Therefore, modulation of apoptosis is a key target for cancer treatment. Apoptosis, a classic form of programmed cell death (PCD), is characterized by an energy-dependent biochemical mechanism, with specific morphological features in which caspase activation plays a significant role. Metformin was discovered to upregulate heat shock protein family A member 5 (HSPA5, Bip), DNA damage-inducible transcript 3 (DDIT3, CHOP), and caspase-12 and induce endoplasmic reticulum stress and endoplasmic reticulum stress-associated apoptosis in vitro and in vivo in thyroid cancer [117]. He et al. [118] proposed that metformin induces intrinsic apoptosis (but not extrinsic apoptosis) of oral squamous cell carcinoma (OSCC) cells in vitro and in vivo. Combined treatment with metformin and 4SC‐202 synergistically upregulated molecules involved in intrinsic apoptosis, including p53, Bax, cleaved caspase‐9, cleaved caspase‐3, and cleaved PARP, and downregulated Bcl‐2, while the key component in extrinsic apoptosis (caspase‐8) was not affected. Lindsay et al. [119] reported that metformin only induces apoptosis in human papillomavirus (HPV)-positive head and neck cancer cells; however, the relationship between HPV oncoproteins and metformin has not been sufficiently explored.

##### Autophagy

Autophagy is a lysosome-dependent process of cellular degradation that removes unnecessary or dysfunctional components. Although traditionally characterized as a degradation pathway to protect against starvation, autophagy has also been found to play an essential role in the homeostasis of nonstarved cells. Metformin increases autophagy-related LC3-II and induces autophagy but does not induce significant apoptosis in myeloma [105]. Furthermore, autophagy and apoptosis can coexist in several types of cancer. Metformin induces apoptosis and autophagy via different mechanisms in osteosarcoma stem cells (OSCs) [104]. Specifically, metformin induces apoptosis via a ROS-mediated mitochondrial dysfunction pathway and regulates autophagy by activating the AMPK/mTOR signaling pathway. Furthermore, metformin-mediated autophagy regulates the homeostasis of stemness and pluripotency in OSCs. Li et al. [63] have also demonstrated that metformin may induce apoptosis and autophagy in osteosarcoma cells. Metformin decreases MMP (ΔΨm), stimulates the cleavage of caspase-3 and PARP, reduces the expression of Bcl-2, and induces apoptosis. Additionally, metformin upregulates LC3B-II, p62, and Beclin-1 levels and induces autophagy. Finally, metformin induces apoptosis and autophagy by activating the ROS-dependent JNK/c-Jun cascade. Feng et al. [64] discovered an interaction between apoptosis and autophagy in esophageal squamous cell carcinoma (ESCC) cells in which the inhibition of autophagy sensitizes ESCC cells to metformin-induced apoptosis.

However, in other settings, autophagy does not affect the cell death process. Babcook et al. [109] reported that the combined treatment of simvastatin and metformin upregulates autophagy in C4-2B osseous metastatic CRPC cells because of chemoresistance but does not play a part in the cell death process.

##### Pyroptosis

Pyroptosis, a highly inflammatory form of lytic programmed cell death, can be considered an alternative treatment for some cancers resistant to apoptosis. Metformin has been shown to induce pyroptosis in ESCC by targeting the miR-497/PELP1 axis in vitro and in vivo [99]. The pyroptosis induced by metformin is mediated by gasdermin D (GSDMD) and abrogated by the forced expression of PELP1. Zheng et al. [120] reported that metformin treatment activates AMPK/SIRT1/NF-κB signaling to induce caspase3/GSDME-mediated cancer cell pyroptosis. Furthermore, mitochondrial dysfunction plays a role in metformin-induced pyroptosis in cancer cells.

##### Necrosis

Necrosis has been understood as unprogrammed cell death. Necroptosis has recently been suggested to be a regulated form of necrosis. Babcook et al. [109] reported that the combination of simvastatin and metformin upregulates Ripk1 and Ripk3 protein expression, necrosome formation, HMGB-1 extracellular release, necrotic induction, and viability rescue with necrostatin-1- and Ripk3-targeting siRNA. This combination does not lead to apoptosis but instead necrosis dependent on Ripk1 and Ripk3 in C4-2B osseous metastatic CRPC cells. Necrosis is a potential target in apoptosis- and chemotherapy-resistant cancer cells.

##### Ferroptosis

Ferroptosis is a newly defined type of iron-dependent programmed cell death. It is characterized by the accumulation of lipid peroxides and is genetically and biochemically distinct from other forms of regulated cell death. Metformin induces ferroptosis in an AMPK-independent manner to inhibit breast cancer growth [121]. Mechanistically, metformin decreases the stability of SLC7A11, a key regulator of ferroptosis, by inhibiting its UFMylation.

However, Alimova et al. [122] found that metformin did not induce apoptosis in breast cancer cells. Similarly, Mogavero et al. [112] reported that while metformin was cytostatic and decreased cell motility, it did not induce cell death, including apoptosis, autophagy, or senescence, in colorectal cancer cells. Moreover, its effects on cells were found to be reversible through drug discontinuation.

Overall, metformin alone or in combination with other agents has the potential to regulate various types of cell death via diverse pathways.

### Senescence

Cellular senescence is a stable state of cell cycle arrest that promotes tissue remodeling during normal development and when tissue is damaged [123]. In general, cell senescence, which acts as a tumor-suppressor mechanism, can irreversibly arrest the growth of cells at risk of neoplastic transformation [124]. Metformin was reported to inhibit cancer by inducing the senescence of several cancer cells [125]. The activation of AMPK, the AMPK-SIRT1 pathway [126] or p53 [125, 127] is required in metformin-induced senescence. In addition, metformin can lower the threshold for stress-induced senescence to generate a “stressed” cell phenotype that becomes presensitized to oncogenic-like stimuli such as DNA damage and proliferative and/or stemness inducers [128]. Additionally, metformin cooperates with other agents during senescence. The anticancer effects of CDK4/6 inhibitors can be enhanced by metformin by reprogramming the profiles of the senescence-associated secretory phenotype (SASP) [129]. The addition of metformin following androgen-deprivation therapy can induce apoptosis, attenuate mTOR activation, and reduce the number of senescent cells in prostate cancer [130].

However, metformin has also been reported to help cancer cells evade senescence. Hoppe-Seyler et al. [131] reported virus/host cell crosstalk in human papillomavirus (HPV)-positive cancer cells. Although metformin suppresses the HPV oncogene by downregulating cellular factors associated with E6/E7 expression, it only induces a reversible discontinuation of proliferation in HPV-positive cancer cells, helping them evade senescence. Metformin also effectively blocks senescence induced by E6/E7 inhibition or chemotherapy in HPV-positive cancer cells.

### Locking phenotypic plasticity

Cancer stem cells (CSCs), a cluster of tumor cells possessing clonogenicity and self-renewal abilities, may play a role in tumor recurrence and metastasis. Metformin has been shown to be preferentially cytotoxic to CSCs compared to non-CSCs [132]. Clonal cell growth and cancer sphere formation are hallmarks of CSCs that can be inhibited by metformin. Metformin has been reported to suppress the expression of CSC markers, including CD44, EpCAM, EZH2, Notch-1, Nanog, and Oct4 in pancreatic cells [133]; CD44 and Sox2 in gastric cancer [110]; Nanog, c-Myc, and TLF4 in NSCLC [62]; and upregulate the expression of differentiation markers, such as Kruppel-like factor 4 (KLF4) and MUC5AC in gastric cancer [110]. Metformin suppresses the self-renewal ability and tumorigenicity of osteosarcoma stem cells via ROS-mediated apoptosis and autophagy [104].

### Inspiring inflammation and immunity in cancer (Fig. 4)

**Fig. 4:**
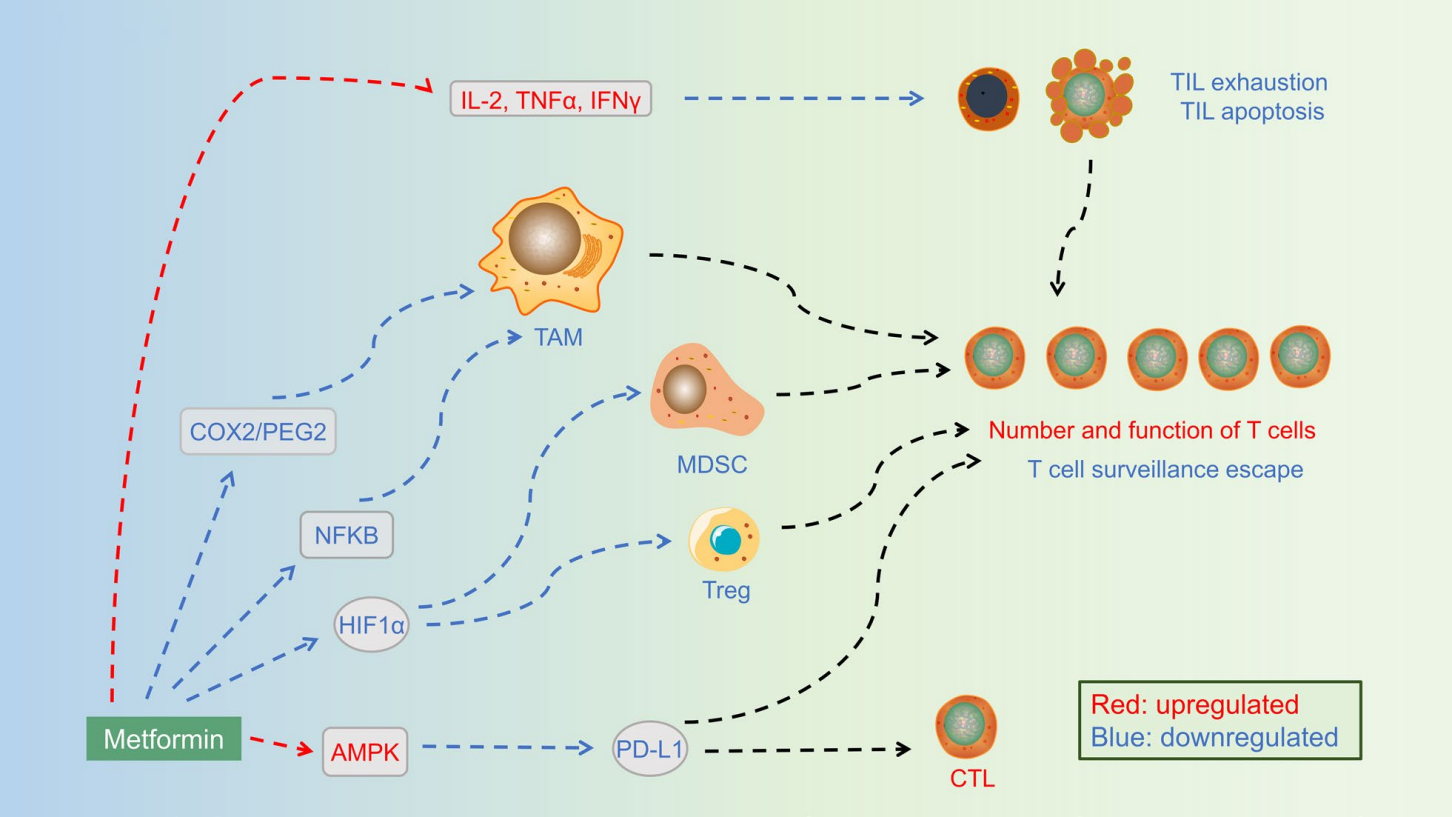
Metformin regulates diverse factors to modulate immune cells in the tumor microenvironment to inspire immunity in cancer. Metformin can modulate tumor infiltrating lymphocytes (TIL), tumor-associated macrophages (TAMs), Treg, myeloid-derived suppressor cells (MDSCs), and PDL1 to increase the number and function of T cells and decrease T cell surveillance escape. Metformin can also downregulate PDL1 to increase cytotoxic T cells

Breakthroughs in cancer immunotherapy have expanded the possibilities for cancer therapy over the last decade. Although cancer immunity has continued to be underappreciated, an increasing number of studies have focused on the relationship between cancer, immunity, and potential immunotherapy. Metformin has been found to interact with immune regulators, such as inhibitory immune checkpoints, M2-like tumor-associated macrophages (TAMs), regulatory T cells (T-regs), and myeloid-derived suppressor cells (MDSCs), to inhibit immune destruction.

CD8^+^ tumor-infiltrating lymphocytes (TILs) inevitably undergo immune exhaustion, which is characterized by decreased production of multiple cytokines, such as IL-2, TNFα, and IFNγ, followed by a reduction in apoptosis. Metformin increases CD8^+^ TILs and protects them from exhaustion and apoptosis in the TME. Furthermore, the adoptive transfer of metformin-treated antigen-specific CD8+ T cells efficiently migrates into tumors and maintains multifunctionality in a manner that is sensitive to the AMPK inhibitor compound C [134]. Metformin also inhibits TAM infiltration during prostate cancer initiation and progression by inhibiting the COX2/PGE2 axis [135].

A window of opportunity trial for HNSCC has demonstrated that metformin modulates metabolism in the HNSCC microenvironment [136]. Metformin decreases infiltration of FOXP3+ T regulatory cells in intratumor regions, increases CD8+ cytotoxic T cell infiltration in the peritumoral leading edge stroma, and increases the CD8/FOXP3 ratio both in the tumor and leading-edge stroma of primary HNSCC tumors [137]. Metformin may positively interact with the immune TME in HNSCC, regardless of HPV status. Metformin inhibits CCR1 surface expression in HNSCC cells and the expression of CCL15 in M2-type TAMs, which promote HNSCC cell resistance to gefitinib under hypoxic conditions through the CCL15-CCR1-NF-κB pathway [138].

In a zebrafish model of nonalcoholic fatty liver disease (NAFLD)-associated hepatocellular carcinoma, metformin was found to alter macrophage polarization and exacerbate the liver inflammatory microenvironment and cancer progression [139]. In addition, metformin rescued the effects of a high-fat diet (HFD) on liver tumorigenesis (angiogenesis, steatosis, lipotoxicity), inflammation, and T cell recruitment to the liver.

Combined with immune checkpoint blockade (ICB), metformin has been found to decrease T-reg and MDSC levels and increase CD8+ levels in murine models [140]. Notably, only long-term metformin treatment is sufficient to reduce cancer cell growth. Programmed cell death 1 (PD-1)/programmed death ligand 1 (PD-L1), a representative ICB, has initiated a new era in cancer treatment. Cha et al. [141] reported that PD-L1 expression decreases with AMPK activation in tumor tissues taken from metformin-treated breast cancer. AMPK activation by metformin phosphorylates S195 and PD-L1, subsequently inducing abnormal PD-L1 glycosylation, leading to endoplasmic reticulum accumulation and endoplasmic reticulum-associated degradation. Metformin also increases cytotoxic T lymphocyte (CTL) activity by reducing the stability and membrane localization of PD-L1. Wen et al. [142] reported that metformin enhances the membrane dissociation of the cytoplasmic domain of PD-L1 (PD-L1-CD) by disrupting electrostatic interactions, thus decreasing the cellular abundance of PD-L1.

### Polymorphic microbiome

There is evidence that metformin can alter the microbiota community and regulate human metabolism [143–146]. Recently, some reports revealed the relationship between gut microbiota regulation by metformin and cancer. Dong et al. revealed that metformin alters the duodenal microbiome and decreases the incidence of pancreatic ductal adenocarcinoma facilitated by diet-associated obesity [147]. Huang et al. also reported that metformin modulates the gut microbiota and rescues Fusobacterium nucleatum-induced colorectal tumorigenesis in experimental animals [148].

## Clinical trials

Numerous clinical trials have been conducted in the pursuit of more convincing evidence of metformin’s anticancer effects and to determine the appropriate anticancer uses of metformin. Here, we have included 34 completed clinical trials with results available on the clinicaltrials.gov website among the 182 results searched until March 2023 (Table 1). These include brain tumors [149], HNSCC [15, 22, 136, 137], acute lymphoblastic leukemia [150], and breast [76, 77, 151–157], lung [158], esophageal [136, 159], colorectal [160, 161], pancreatic [162–164], endometrial [165–169], ovarian [14, 170], and prostate [13, 171–175] cancers. Among them, two trials are in phase 3, and 16 trials combine metformin with chemotherapy and/or radiotherapy and/or targeted therapy.

**Table 1:** Clinical trials of MET application in cancers

Study design	Study population	Dosing regimen	Results	Ref.
Phase 2, single group assignment, open label, for basic science	• Breast cancer • 41 participants enrolled	Arm: MET (extended release MET 1500 mg once daily for 14–21 days)	Mitochondrial response to MET may define anti-tumor effect MET reduces fatty acid oxidation with a subsequent accumulation of intracellular triglycerides, independent of AMPK activation	[76, 77]
Phase 2, randomized, parallel assignment, double blinded, for prevention	• Premenopausal, overweight or obese women with metabolic disturbances • 151 participants enrolled	Arm: MET (850 mg QD * 4 weeks, 850 mg BID * 12 months) Arm: placebo	MET did not change percent breast density and dense breast volume but led to a numerical but not significant decrease in non-dense breast volume	[151]
Randomized, factorial assignment, quadruple blinded, for prevention	• Breast cancer • 333 participants enrolled	Arm: MET (500 mg * week 1; 1000 mg * weeks 2–4 + 1500 mg * weeks 5+) + lifestyle intervention Arm: placebo + lifestyle intervention	In non-cancer patients, 6 months of MET therapy may be inadequate to observe expected epigenetic age deceleration	[152]
Single group assignment, open label, for treatment	• Breast cancer • 39 participants enrolled	Arm: MET (500 mg TID * 2–3 weeks after diagnostic biopsy until surgery	MET reduced PKB/Akt and ERK1/2 phosphorylation, coupled with a decrease in insulin and IR levels, suggesting insulin-dependent effects are important in the clinical setting	[188]
Phase 2, randomized, parallel assignment, quadruple blinded, for treatment	• Non-diabetic metastatic breast cancer • 40 participants enrolled	Arm: placebo + chemotherapy Arm: MET (850 mg BID) + chemotherapy	The addition of MET to standard chemotherapy showed no significant effect on response rate, PFS, or OS but was associated with increased grade I and II adverse events and decreased global quality of life	[153]
Phase 2, single group assignment, open label, for treatment	• HER-2-positive breast cancer • 49 participants enrolled	Arm: liposomal doxorubicin + docetaxel + trastuzumab + MET (single agent day − 13 to day 0; 1000 mg QD day − 13 to − 11; 1000 mg BID from day − 10)	The concomitant administration of trastuzumab, liposomal doxorubicin, docetaxel, and MET was safe and showed good activity, but did not appear to improve activity over conventional sequential regimens	[154]
Phase 2, randomized, parallel assignment, open label, for treatment	• HER2 negative metastatic breast cancer • 126 participants enrolled	Arm: MET (1000 mg QD * day 1–3; 1000 mg BID after) + Myocet + cyclophosphamide Arm: Myocet + cyclophosphamide	The results excluded any beneficial effect of MET in combination with chemotherapy either in terms of PFS or OS	[155]
Phase 3, randomized, parallel assignment, triple blinded, for treatment	• Breast cancer • 3649 participants enrolled	Arm: MET hydrochloride (QD * week 1–4, BID * 5 years) Arm: placebo	MET statistically significantly improved weight, insulin, glucose, leptin, and CRP at six months. Effects did not vary by baseline BMI or fasting insulin Among patients with high-risk operable breast cancer without diabetes, the addition of MET did not significantly improve invasive disease-free survival	[156, 157]
Phase 2, randomized, parallel assignment, open label, for treatment	• Stage III NSCLC • 170 participants enrolled	Arm: radiation therapy + carboplatin + paclitaxel Radiation therapy + carboplatin + paclitaxel + MET (500 mg BID * days 1–7, 500 mg TID * days 8–14, 500 mg, 1000 mg, and 500 mg TID * days 15–126)	The addition of MET to chemoradiation was well-tolerated but did not improve OS or PFS	[176]
Phase 2, randomized, parallel assignment, double blinded, for treatment	• EGFR-mutation-positive NSCLC • 224 participants enrolled	Arm: gefitinib + MET (500 mg BID * 1 week, 1000 mg, 500 mg BID * 1 week, then 1000 mg BID) Arm: gefitinib + placebo	Addition of MET did not show enhanced gefitinib efficacy and hence this study does not support the concurrent use of MET with first-line EGFR-TKI therapy in non-diabetic EGFRm NSCLC patients	[158]
Phase 3, randomized, crossover assignment, quadruple blinded, for treatment	• Brain tumor treated with cranial or cranial-spinal radiation • 24 participants enrolled	Arm: MET (500 mg/m^2^ daily given in 2 doses * 1 week, 1000 mg/m^2^ daily given in 2 doses * 12 weeks) Arm: placebo	MET was linked to better performance on tests of declarative and working memory The effects of MET on cognition and brain structure are feasible and MET is safe and tolerable	[149]
Early phase 1, single group assignment, open label, for basic science	• Head and neck squamous cell cancer • 50 participants enrolled	Arm: MET (2000 mg per day * ≥ 9 days prior to surgery	MET increases markers of reduced catabolism and increases senescence in stromal cells and cancer cell apoptosis MET may favorably alter the immune TME independent of HPV status MET mediates immune antitumorigenic function through NK cell-mediated cytotoxicity and downregulation of CXCL1	[22, 136, 137]
Phase 1, single group assignment, open label, for treatment	• Head and neck cancer • 20 participants enrolled	Arm: MET (including 2000 mg, 2550 mg, and 3000 mg daily in divided doses) + drug: cisplatin + radiation therapy	Rates of OS and PFS were encouraging in this limited patient population	[15]
Phase 2, randomized, parallel assignment, double blinded, for prevention	• Barrett esophagus esophageal cancer • 93 participants enrolled	Arm: MET hydrochloride (extended-release tablet PO QD * week 1, BID * weeks 2–12) Other: placebo	MET did not cause major reductions in esophageal levels of pS6K1 and did not discernibly alter epithelial proliferation or apoptosis in esophageal tissues	[159]
Phase 2, parallel assignment, quadruple blinded, for treatment	• Familial adenomatous polyposis • 34 participants enrolled	Arm: MET (500 mg QD) Arm: MET (1500 mg QD) Arm: placebo	Seven months of treatment with 500 mg or 1500 mg metformin did not reduce the mean number or size of polyps in the colorectum or duodenum in familial adenomatous polyposis patients	[160]
Phase 2, single group assignment, open label, for prevention	• Colorectal adenomas and obesity • 45 participants enrolled	Arm: MET hydrochloride (500 mg extended release tablets QD * week 1, with a dose escalation of 500 mg each week until the final dose of 1000 mg BID week 4–12)	MET showed no significant change in activated S6serine235	[161]
Phase 1, randomized, parallel assignment, open label, for treatment	• Metastatic pancreatic adenocarcinoma • 22 participants enrolled	Arm: MET (850 mg BID on a 28 day cycle) Arm: MET + rapamycin	MET ± rapamycin maintenance for mPDA was well-tolerated and several patients achieved stable disease associated with exceptionally long survival	[162]
Phase 2, single group assignment, open label, for treatment	• Gemcitabine-refractory advanced pancreatic adenocarcinoma • 41 participants enrolled	Arm: paclitaxel + MET (850 mg Q8H)	Despite the encouraging pre-clinical evidence of antitumor activity of MET, the primary endpoint disease control rate was not met The combination was poorly tolerated	[163]
Phase 2, randomized, parallel assignment, quadruple blinded, for treatment	• Locally advanced pancreatic cancer, metastatic pancreatic cancer • 120 participants enrolled	Arm: gemcitabine + erlotinib + MET (increased from 500 mg BID in week 1 to 1000 mg BID in week 2) Arm: gemcitabine + erlotinib + placebo	Addition of MET did not improve outcomes in patients with advanced pancreatic cancer treated with gemcitabine and erlotinib	[164]
Early phase 1, single group assignment, open label, for treatment	• Endometrial cancer • 21 participants enrolled	Arm: MET (850 mg QD * ≥ 7 days, up to 30 days before surgery)	MET decreased serum levels of IGF-1, omentin, insulin, C-peptide, and leptin between pre- and post-treatment samples, as well as decreased phosphorylation of AKT and MAPK	[165]
Early phase 1, single group assignment, open label, for treatment	• Endometrial cancer • 28 participants enrolled	Arm: MET (850 mg QD)	JPT1 represents a predictive and pharmacodynamic biomarker of MET response	[166]
Randomized, parallel assignment, double blinded, for treatment	• Endometrial cancer • 50 participants enrolled	Arm: MET hydrochloride (850 mg QD * ≥ 7 days before surgery) Arm: placebo	Short-term treatment with MET significantly reduced the proliferative marker Ki-67 index in women with endometrioid endometrial cancer awaiting surgical staging	[167]
Phase 2, randomized, parallel assignment, open label, for treatment	• Endometrial atypical hyperplasia • Well-differentiated endometrial adenocarcinoma • 150 participants enrolled	Arm: megestrol acetate and MET (500 mg TID * 3 months) Arm: megestrol acetate	As a fertility-sparing treatment, MET plus MA was associated with a higher early CR rate compared with MA alone in patients with atypical endometrial hyperplasia	[168]
Phase 2, randomized, parallel assignment, open label, for treatment	• Complex endometrial hyperplasia with atypia • Grade 1 endometrial endometrioid adenocarcinoma • 165 participants enrolled	Arm: levonorgestrel IUD + MET (500 mg BID) Arm: levonorgestrel IUD drug + behavior: levonorgestrel IUD + weight loss intervention	Complete response rates at 6 months were encouraging for patients with endometrial adenocarcinoma and endometrial hyperplasia with atypia across the three groups	[169]
Phase 1, single group assignment, open label, for treatment	• Epithelial ovarian cancer • 15 participants enrolled	Arm: MET + carboplatin + paclitaxel	The recommended phase II dose of MET in combination with carboplatin and paclitaxel in advanced ovarian cancer is 1000 mg TID	[170]
Phase 2, single group assignment, open label, for treatment	• Ovarian, fallopian tube, and primary peritoneal cancer • 90 participants enrolled	Arm: MET (a) Neoadjuvant MET, debulking surgery, and adjuvant chemo plus MET (b) Neoadjuvant chemo and MET, interval debulking surgery, and adjuvant chemo plus MET	MET impacted epithelial ovarian cancer CSCs and was associated with better-than-expected OS	[14]
Phase 2, randomized, parallel assignment, double blinded, for treatment	• Pre-prostatectomy prostate cancer • 20 participants enrolled	Arm: MET hydrochloride (extended-release MET hydrochloride QD for 4–12 weeks before surgery) Arm: placebo	MET distributes into human prostate tissue, suggesting that MET could exert its effects directly on tissue targets Concomitant MET with radiotherapy and androgen deprivation therapy was associated with inferior biochemical outcome	[171, 189]
Phase 2, single group assignment, open label, for treatment	• Metastatic castration-resistant prostate cancer • 25 participants enrolled	Arm: MET (1000 mg BID in uninterrupted 4-week cycles) + abiraterone	The addition of MET to abiraterone does not affect further progression and has no meaningful clinical benefit A higher-than-expected gastrointestinal toxicity attributed to MET was observed	[173]
Phase 2, randomized, parallel assignment, open label, for treatment	• Overweight or obese prostate cancer patients • 29 participants enrolled	Arm: observation and bicalutamide Arm: MET (1000 mg BID) and bicalutamide	Although MET plus bicalutamide was well tolerated, there was no improvement in rates of achieving undetectable PSA at 32 weeks. MET monotherapy induced modest PSA declines. MET, given alone and in combination, displayed immune modifying effects, primarily within NK and T cells subsets	[174]
Phase 2, single group assignment, open label, for treatment	• Chemotherapy-naïve castration-resistant prostate cancer • 44 participants enrolled	Arm: MET (1000 mg BID)	Treatment with MET is safe in non-diabetic patients, and it yields objective PSA responses and may induce disease stabilization	[190]
Phase 2, randomized, parallel assignment, quadruple blinded, for treatment	• Metastatic hormone-refractory prostate cancer • 100 participants enrolled	Arm: placebo Arm: MET (850 mg BID)	MET addition failed to improve the standard Docetaxel regimen in metastatic castration-resistant prostate cancer	[175]
Phase 1, randomized, parallel assignment, open label, for treatment	• Solid tumor • 105 participants enrolled	Arm: MET + chemotherapy	Post-MET increase in AMPK phosphorylation may potentially explain lack of disease progression in nearly half of our patients MET can be given safely with chemotherapy	[21]
Phase 2, randomized, parallel assignment, single blinded, for prevention	• Malignant solid tumor • 121 participants enrolled	Arm: MET (2,000 mg per day * 12 months) Behavioral: self-control weight loss Arm: coach directed behavioral weight loss	MET treatment did not show significant changes in serum urate compared to baseline MET impacted gut microbiota composition, altered circulating short-chain fatty acids, including increasing acetate, which correlated with lower fasting insulin Similar weight loss was observed in the behavioral weight loss arm and the MET arm	[191–193]
non-randomized, parallel assignment, open label, for basic science	• Acute lymphoblastic leukemia • 102 participants enrolled	Arm: MET (850 mg TID + prednisone * 7 days and * 28 days)	MET was a protective factor against both therapeutic failure and early relapse The combined use of MET with chemotherapy is effective in patients with elevated levels of ABCB1 gene expression	[150]

*MET* metformin, *QD* once a day, *BID* twice a day, *IR* insulin receptor, *PFS* progression-free survival, *OS* overall survival, *NSCLC* non-small cell lung cancer, *mPDA* metastatic pancreatic ductal adenocarcinoma, *JPT1* Jupiter microtubule-associated homolog 1, *MA* megestrol acetate, *IUD* intrauterine device, *TID* three times a day, *CSC* cancer stem cell

The results varied in biochemical indicators, PFS, OS, and adverse events. A double-blind, placebo-controlled crossover pilot study revealed that metformin was linked to better performance in the assessment of cognitive and neural recovery, with good safety and tolerance in survivors of pediatric brain tumors [149]. A multicenter phase 2 trial reported that metformin induced objective PSA responses and disease stabilization in patients with chemotherapy-naive castration-resistant prostate cancer without diabetes [13]. However, some clinical trial results did not support the application of metformin in cancer treatment. A triple-blinded, randomized, placebo-controlled phase 3 trial with 3649 participants reported that the addition of metformin did not significantly improve invasive disease-free survival among patients with high-risk operable breast cancer without diabetes [157]. A double-blind, randomized, placebo-controlled phase 2 trial reported that the addition of metformin to gemcitabine and erlotinib did not improve outcomes in patients with advanced pancreatic cancer [164]. Another phase 2 randomized clinical trial showed that the addition of metformin to chemoradiation was well tolerated but did not improve the OS or PFS of patients with stage III NSCLC [176]. A randomized, double-blind, phase 2 trial even discovered that the addition of metformin to gefitinib resulted in nonsignificantly worse outcomes and increased toxicity in NSCLC patients without diabetes harboring EGFR mutations [158]. Besides, there are no synergetic benefits of metformin and chemotherapy or antiandrogen therapy in prostate cancer patients [174, 175]. However, the clinical trials had some limitations, such as a lack of accurate molecular stratification of patients, insufficient compliance of patients and a limited study population. Hopefully, more well-designed clinical trials are being conducted, including phase 3 multicenter studies with participants with or without diabetes enrolled, and they may provide more convincing evidence regarding the anticancer efficacy and safety of metformin alone or in combination with chemoradiotherapy, targeted therapy or immunotherapy.

## Discussion

The repurposing of metformin has always been a research hotspot worldwide. Currently, the benefits of metformin to aging-related diseases have gained more importance since the speedup of aging in our society. Metformin was reported to mimic significant metabolic effects of caloric restriction, which was the only acknowledged strategy to robustly extend health and lifespan in mammals [177]. For cancer, an aging-related disease, metformin exhibited promising anticancer effects in preclinical studies. Thus, the anticancer mechanisms of metformin have received considerable attention. Metformin inhibits mitochondrial complex I and triggers energy depletion, which activates AMPK and inhibits mTOR, restraining cancer growth via the maintenance of energy homeostasis. Besides, metformin also exerts anticancer effects that are independent of AMPK but rather dependent on Rag GTPases or REDD1. Moreover, some additional anticancer pathways involve the IGF-1R/PI3K/AKT/mTOR pathway and the p53 pathway. Intriguingly, metformin can exert influences on the hallmarks of cancer, including regulation of the cell cycle, cell death, CSCs, cancer cell migration, invasion and metastasis, cancer metabolism, and cancer immunity. In any case, the specific mechanism remains to be clarified, and the explicit anticancer mechanisms of metformin should be further investigated.

For basic research on the molecular mechanisms of metformin in anticancer therapy, we consider that the main challenges lie in three aspects: (1) finding the direct cellular targets of metformin that mediate anticancer activities; (2) clarifying the key questions of the “direct effects” and the “indirect effects” as well as which one plays a greater role in anticancer actions; and (3) developing in vivo models that can mimic the indirect and direct effects of metformin. Many endeavors have been devoted to solving these problems. First, the latest studies have managed to explore the direct cellular targets of metformin. For example, Ma et al. [40] revealed that PEN2 is the direct molecular target of metformin using a photoactive metformin probe. Bridges et al. [38] recently defined the inhibitory drug-target interactions of metformin with mammalian respiratory complex I by combining cryo-electron microscopy and enzyme kinetics. The identification of the direct target of metformin’s anticancer effects may help to further investigation for drug development. Second, in in vitro research, the direct effects are emphasized and well-studied, while the indirect effects cannot be mimicked. Some in vivo and clinical studies suggest that indirect insulin-dependent effects may be of great significance in at least some cancers, such as breast cancer and lung cancer. The research directions varied for the two modes of anticancer effects (Table 2). In terms of “direct effects,” more effort should be put into markers such as LKB1, Rag GTPases and REDD1. In terms of “indirect effects,” more effort should be put into markers such as blood glucose and insulin levels, insulin resistance, expression of insulin receptors and insulin-like growth factor receptor 1, and targets in the liver. Understanding the markers helps to predict the therapeutic response of patients. For example, the synergistic effects between metformin and gefitinib were reported to rely on the presence of wild-type LKB1 in NSCLC cells [178]. Third, since indirect effects could not be simulated in vitro, more in vivo models are needed to reexamine the direct and indirect effects of metformin and the possible interactions. Some attempts have been made to develop related models. For instance, in research on the anticancer activity of metformin, hyperglycemic mice were reported to lose sensitivity to metformin compared with normoglycemic mice, probably through increased c-Myc expression, glycolytic enzymes hexokinase 2 and pyruvate dehydrogenase kinase 1 [179]. We expect more in vivo models with more complex designs that are currently used in most in vitro research.

**Table 2:** Markers with potential predictive value

Mechanism	Markers
Direct effects	LKB1
	Rag GTPases
	REDD1
	mTOR
Indirect effects	Blood glucose level
	Insulin/fasting insulin level
	Insulin resistance
	Insulin receptors/insulin-like growth factor receptor 1
	Targets in the liver (e.g., OCT1/2/3 expression)

There is a discrepancy in the antitumor effect of metformin between clinical research and preclinical studies, although metformin has shown notable benefits for cancer prevention and treatment in preclinical research, and the related molecular mechanisms have been extensively studied. The challenges mainly include (1) simulating pharmacokinetics consistent with clinical settings, including appropriate metformin concentrations and dosing time; (2) exploring suitable synergetic therapies and patients who are more sensitive to metformin; and (3) utilizing other forms of biguanides, such as phenformin or modified biguanides, which have better performances. Therefore, further research is required regarding the critical aspects mentioned above. First, many preclinical studies have employed metformin at concentrations that are considerably higher than what would be deemed safe in clinical settings [180]. The plasma concentrations of metformin were reported to be 5–30 μmol/L in patients taking clinical doses of 1.5–2 g per day, which were the most common doses for diabetes and were used in most clinical trials for cancer treatment [181]. However, the concentrations in most preclinical studies in vitro (300 μmol/L–10 mmol/L) were dozens or even a thousand times the clinical concentrations. The dosages of in vivo studies (200–1000 mg/kg per day) were 6–30 times the clinical dosages (approximately 30 mg/kg per day) with metformin diluted in the drinking water or intraperitoneally injected. Recent studies have noted this issue. Metformin can suppress cancer at a clinically safe concentration in vitro [40] and in vivo [16, 35, 65]. A recent report also proposed that AMPK activation by clinical concentrations of metformin was not completely the consequence of changes in the cellular AMP/ATP ratio or depletion of cellular energy charge [182]. For future research, it is possible to reasonably expand the metformin concentration and set more concentration gradients. Besides, metformin tends to be administered at the very beginning of preclinical models, which cannot be replicated in clinical settings. For example, a synergetic therapy of metformin and PD1 blockade for melanoma was reported to have a tumor size threshold. Once tumors were larger than 10 mm^2^ before treatment in mice, metformin failed to exert synergistic effects with PD-1 blockade [183]. In the clinical setting, improved outcomes were observed only among patients with early-stage NSCLC or those who took metformin before the NSCLC diagnosis [184]. Therefore, it enlightens us to apply metformin once cancer is diagnosed or even in people with a high risk of cancer if possible. Second, based on our understanding of metformin’s effects on cancer hallmarks, metformin could be a useful adjuvant agent in combination therapy to combat cancer synergistically in certain patients with certain cancers [36]. It can be administered along with chemotherapy, radiotherapy, immunotherapy or targeted therapy. Taking immunotherapy as an example, a high level of lactate can lead to tumor immune tolerance, while metformin was reported to increase the level of lactate in the intra- and extracellular environment [31, 185]. However, from the current evidence, acidification of the TME made tumors more susceptible to metformin due to the loss of NAD+ regeneration capacity [78, 79, 186]. Therefore, whether metformin can cause tumor immune tolerance by increasing the acidification of the TME remains an interesting issue to explore. If indeed, the combination of metformin and immunotherapy might be a possible direction in further research. Besides, metformin was reported to show no more benefits in some cancers with certain mutations [158] or advanced stages [164, 175, 176] or in patients without diabetes [157, 158]. We call for further high-quality clinical trials on metformin combined with other therapies in different types of physiological conditions and cancers. Third, it was reported that phenformin may outperform metformin owing to its unique pharmacokinetic characteristics, which include better absorption and inhibition of the mitochondria [187]. Although the incidence of lactic acidosis associated with phenformin is higher than that associated with metformin, phenformin is in any case safer than other cancer treatments. Moreover, given the pharmacokinetic differences between metformin and phenformin, we can obtain more insights regarding drug modification. Once there is more evidence, we expect metformin or other forms of biguanides to exert a greater influence on anticancer therapy, at the appropriate dosage, on patients of appropriate metabolic state, and in combination with other therapies.

## Conclusion

The review details the possible molecular mechanisms of metformin in cancer prevention and treatment, elucidates its role in terms of cancer hallmarks, and more importantly, analyses current challenges and future directions in clinical translation.

## Data Availability

All data generated are included in this published article or from public sources.
